# Chitobiose utilization in *Borrelia burgdorferi *is dually regulated by RpoD and RpoS

**DOI:** 10.1186/1471-2180-9-108

**Published:** 2009-05-27

**Authors:** Ryan G Rhodes, Wendy Coy, David R Nelson

**Affiliations:** 1 Department of Cell and Molecular Biology, University of Rhode Island, Kingston, RI 02881, USA; 2 Department of Biological Sciences, University of Wisconsin-Milwaukee, Milwaukee, WI 53201 USA

## Abstract

**Background:**

*Borrelia burgdorferi *has limited biosynthetic capabilities and must scavenge N-acetylglucosamine (GlcNAc), an essential component of the microbial cell wall, from the surrounding environment. Spirochetes cultured in the absence of free GlcNAc exhibit biphasic growth; however, addition of chitobiose (a dimer of GlcNAc) substitutes for free GlcNAc resulting in a single exponential phase. We evaluated the effect of RpoS and RpoN, the only alternative sigma factors in *B. burgdorferi*, on biphasic growth and chitobiose utilization in the absence of free GlcNAc. In addition, we investigated the source of GlcNAc in the second exponential phase.

**Results:**

By comparing the growth of wild-type cells to insertional mutants for *rpoS *and *rpoN *we determined that RpoS, but not RpoN, partially regulates both biphasic growth and chitobiose utilization. The *rpoS *mutant, cultured in the absence of free GlcNAc, exhibited a significant delay in the ability to initiate a second exponential phase compared to the wild type and *rpoS *complemented mutant. Expression analysis of *chbC*, which encodes the membrane-spanning protein of the chitobiose phosphotransferase system, suggests the delay is due to the inability of the *rpoS *mutant to up regulate *chbC*. Furthermore, supplementing GlcNAc starved cultures with high concentrations (75 or 150 μM) of chitobiose resulted in biphasic growth of the *rpoS *mutant compared to a single exponential phase for the wild type and *rpoS *complemented mutant. In contrast, growth of the *rpoN *mutant under all conditions was similar to the wild type. 5' Rapid amplification of cDNA ends (5' RACE) revealed the transcriptional start site for *chbC *to be 42 bp upstream of the translational start site. Analysis of the *chbC *promoter region revealed homology to previously described RpoD and RpoS *B. burgdorferi *promoters. We also determined that yeastolate, a component of the growth medium (BSK-II), is not essential for second exponential phase growth.

**Conclusion:**

Together these results suggest that RpoD and RpoS, but not RpoN, regulate biphasic growth and chitobiose utilization in *B. burgdorferi *by regulating the expression of the chitobiose transporter (*chbC*). The data also demonstrate that the second exponential phase observed in wild-type cells in the absence of free GlcNAc is not due to free chitobiose or GlcNAc oligomers present in the medium.

## Background

*Borrelia burgdorferi *is the etiologic agent of Lyme disease, the most common vector-borne disease in the United States. The spirochete is maintained in an enzootic cycle, alternating between a tick vector (*Ixodes scapularis*) and vertebrate host. Uninfected larval ticks acquire *B. burgdorferi *after feeding on a vector-competent host, and spirochetes colonize and persist within the tick midgut for months as the tick molts to the nymphal stage [1]. In the infected-unfed tick, *B. burgdorferi *is associated with the midgut epithelium, existing in a non-replicative state in a nutrient poor environment. When infected nymphs begin to feed, the number of spirochetes increases as nutrients required for growth become more abundant [2]. The spirochetes move from the midgut of the feeding tick to the hemolymph and then to the salivary glands where they can be transferred to a naïve host, a process that occurs no earlier than 24 hours after tick attachment [3].

Small rodents or birds are the primary reservoirs of *B. burgdorferi*; however, *I. scapularis *occasionally transmits the bacterium to larger vertebrates, including humans [1]. Upon infection in humans, spirochetes disseminate from the site of inoculation and may move to tissues other than the skin resulting in numerous clinical manifestations [1]. Symptoms of the primary infection are typically observed days to weeks after the tick bite and include flu-like symptoms that may be accompanied by a macular rash known as erythema migrans. If left untreated other symptoms may present months after inoculation, resulting in arthritis, myocarditis, and/or lesions of the peripheral and central nervous systems [1].

While *B. burgdorferi *has evolved to survive in vastly different environments, it has limited biosynthetic capabilities and must obtain most nutrients from its surrounding environment [4,5]. N-acetylglucosamine (GlcNAc) is an essential component of peptidoglycan, the rigid layer responsible for strength of the microbial cell wall. Many bacteria can synthesize GlcNAc *de novo*; however, *B. burgdorferi *must import GlcNAc as a monomer or dimer (chitobiose) for cell wall synthesis and energy. Therefore, *B. burgdorferi *is normally cultured *in vitro *in the presence of free GlcNAc [6].

In the tick much of the GlcNAc is polymerized in the form of chitin, as this is the major component of the tick exoskeleton. In addition, chitin is an integral part of the peritrophic matrix that encases the blood meal during and after tick feeding. This membrane functions as a permeability barrier, enhances digestion of the blood meal, and protects the tick midgut from toxins and pathogens [7]. GlcNAc oligomers released during remodeling of the peritrophic matrix may be an important source of GlcNAc for *B. burgdorferi *in the nutrient limiting environment of the unfed-infected tick midgut [8].

Previous reports have demonstrated that *Borrelia *species cannot reach high cell densities *in vitro *when cultured without free GlcNAc [6,9]. Recent reports by Tilly *et al *[10,11] extended this work in *B. burgdorferi *with three significant findings. First, they observed that spirochetes cultured without free GlcNAc exhibit biphasic growth when the culture is followed for an extended period of time (up to 200 hours). Following an initial log phase, the cells bleb and enter a death phase before recovering and entering a second exponential phase [10]. Second, Tilly *et al *[10] demonstrated that cells cultured without free GlcNAc, but supplemented with chitobiose, exhibit normal growth and reach high cell densities. Based on these results they hypothesized that the second exponential phase might be due to the import of chitobiose via a phosphotransferase system (PTS) encoded by three genes (BBB04, BBB05 and BBB06) on circular plasmid 26 (cp26). Annotation of the genome sequence originally identified this group of genes (*celB, celC *and *celA*) as a cellobiose (dimer subunit of cellulose) transport system. However, functional analysis of BBB04 (*celB*) by Tilly *et al *[10,11] revealed that this group of genes is responsible for the import of chitobiose. Based on these findings they proposed renaming this set of genes, with BBB04 (*celB*), BBB05 (*celC*) and BBB06 (*celA*) now designated *chbC*, *chbA *and *chbB*, respectively [10]. We have adopted this nomenclature for this communication. Finally, Tilly *et al *[11] demonstrated that a *chbC *mutant can be maintained in ticks and mice, and that the mutation of this gene does not affect transmission of spirochetes. While these results suggest that *chbC *is not essential for virulence of *B. burgdorferi*, the studies were conducted in pathogen-free ticks and mice in a controlled laboratory environment. We hypothesize that *chbC *may still play an important role for survival of spirochetes in a natural setting, as ticks are often infected with more than one pathogen [12] and *chbC *may be important for *B. burgdorferi *to compete with other microorganisms to colonize the tick midgut. Therefore, this study was conducted to further investigate the regulation of *chbC*.

Alternative sigma factors are an important mechanism used by many bacteria to regulate gene expression, and can coordinate the expression of multiple genes needed to adapt to a variety of stresses [13]. *B. burgdorferi *encounters differences in temperature, pH and nutrient availability as it cycles between vector and host. Substantial investigation has focused on the differential expression of genes key to colonization, survival, and transmission of spirochetes during its enzootic life cycle [14,15]. Examination of the *B. burgdorferi *genome reveals this organism possesses only two genes that encode for alternative sigma factors, BB0771 (*rpoS*) and BB0450 (*rpoN*) [16]. Studies have demonstrated that these two sigma factors regulate the expression of numerous genes in different environments, and are essential for colonization and survival in both the tick and mammal [17-19]. In this investigation we examine the role of RpoS and RpoN on biphasic growth, the utilization of chitobiose, and the expression of *chbC *in the absence of free GlcNAc.

## Results

### Effect of *rpoS *mutation on growth in BSK-II without GlcNAc

**Figure 1:**
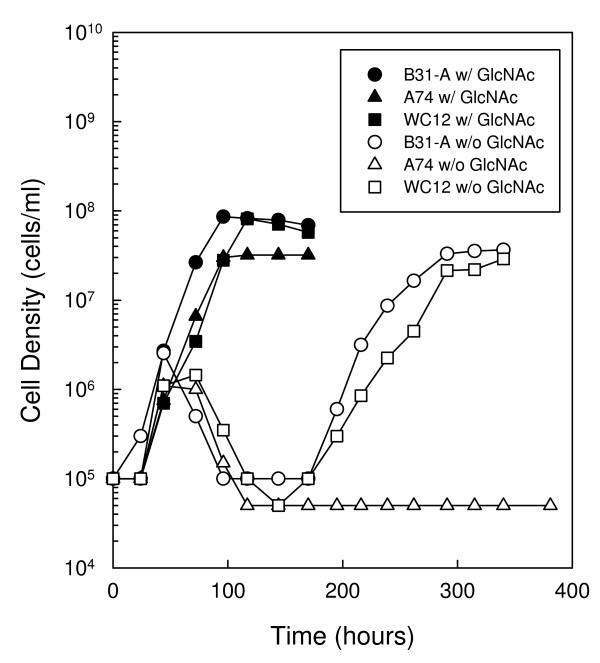
**Mutation of *rpoS *delays biphasic growth during GlcNAc starvation**. Growth of *B. burgdorferi *strains B31-A (WT), A74 (*rpoS *mutant) and WC12 (*rpoS *complemented mutant) in BSK-II with GlcNAc (closed circle, B31-A; closed triangle, A74; closed square, WC12) and without GlcNAc (open circle B31-A; open triangle, A74; open square, WC12). Late-log phase cells from each strain were diluted to 1.0 × 10^5 ^cells ml^-1 ^in the appropriate medium, incubated at 33°C and enumerated daily as described in the Methods. This is a representative experiment that was repeated three times.

**Figure 2:**
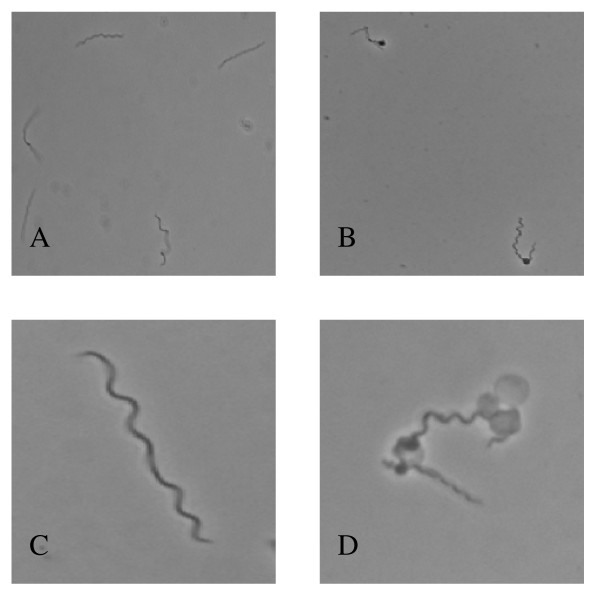
**Morphology of *B. burgdorferi *during GlcNAc starvation**. Phase contrast microscopy of *B. burgdorferi *strain B31-A at 400× (A and B) and 1000× (C and D). Spirochetes were cultured for 72 h in BSK-II with GlcNAc (A and C) and without GlcNAc (B and D).

To determine if the alternative sigma factor, RpoS, is involved in the regulation of biphasic growth, we compared the growth of an *rpoS *mutant strain (A74) to a wild-type strain (B31-A) in the presence and absence of free GlcNAc (Fig. 1). Growth of B31-A and A74 were similar in complete medium, although the wild-type strain reached a slightly higher cell density of 8.6 × 10^7 ^cells ml^-1 ^compared to 3.2 × 10^7 ^cells ml^-1 ^for the *rpoS *mutant. When cells were cultured in the absence of free GlcNAc there was a considerable difference in the ability of the two strains to initiate a second exponential phase. Initially, both strains grew from a starting cell density of 1.0 × 10^5 ^cells ml^-1 ^to ~2.5 × 10^6 ^cells ml^-1 ^by 72 h before entering a death phase characterized by a loss of motility and the formation of blebs near the cell midpoint (Fig. 2B and 2D). As expected, the wild-type strain exhibited biphasic growth, initiating a second exponential phase by 200 h and reaching a peak cell density of 3.65 × 10^7 ^cells ml^-1 ^by 290 h. During the second exponential phase cells exhibited normal morphology characteristic of cells cultured in the presence of GlcNAc (Fig. 2A and 2C). In contrast, the *rpoS *mutant strain did not initiate a second exponential phase by 381 h.

Similar growth experiments were conducted with the *rpoS *complemented mutant, WC12, in an attempt to recover the second exponential phase in A74 (Fig. 1). In complete BSK-II, WC12 showed a growth rate similar to the wild-type and *rpoS *mutant strains, and reached a peak cell density of 8.2 × 10^7 ^cells ml^-1^. When cultured in the absence of free GlcNAc, WC12 exhibited a growth pattern similar to the wild-type B31-A strain. The cells grew to 1.5 × 10^6 ^cells ml^-1 ^by 72 h before entering the characteristic death phase, and then initiated a second exponential phase by 200 h. Taken together, these results suggest that RpoS plays a role in the initiation of the second exponential phase when cells are cultured in the absence of free GlcNAc, possibly due to the regulation of genes important to the process.

### Effect of RpoS on the expression of *chbC*

**Figure 3:**
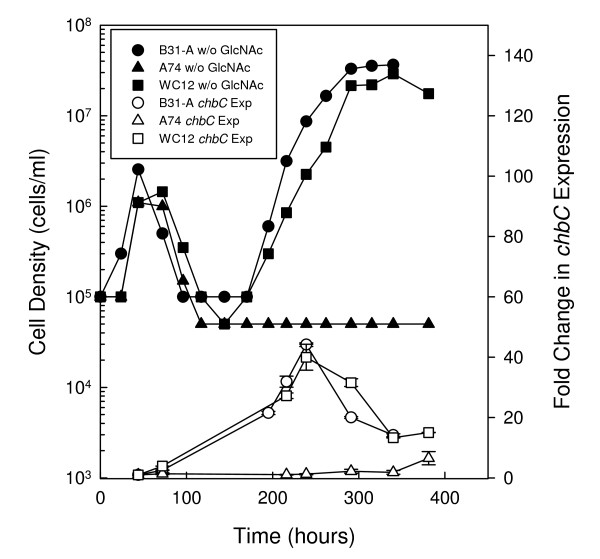
**Mutation of *rpoS *delays the up regulation of *chbC *expression during GlcNAc starvation**. Growth of *B. burgdorferi *strains B31-A (WT), A74 (*rpoS *mutant), and WC12 (*rpoS *complemented mutant) in BSK-II without GlcNAc (closed circle, B31-A; closed triangle, A74; closed square, WC12) and expression of *chbC *transcript in each strain (open circle, B31-A; open triangle, A74; open square, WC12). Late-log phase cells from each strain were diluted to 1.0 × 10^5 ^cells ml^-1^in BSK-II lacking GlcNAc, and RNA was extracted from each strain at various times during growth. Expression of *chbC *was determined by qRT-PCR and the fold change from the initial time point (44 h) was calculated. For expression analyses, duplicate measurements were performed for two biological replicates. Error bars represent the standard error of the mean.

Previously, Tilly *et al *[10] used Northern blot analysis to demonstrate increased expression of *chbC*, the membrane-spanning component of the chitobiose PTS transporter, in the second exponential phase of wild-type cells cultured in the absence of free GlcNAc. They also demonstrated that mutation of the *chbC *gene resulted in a failure of the cells to initiate a second exponential phase by 200 h [10]. From these data they concluded that *chbC *expression is critical for initiation and growth of *B. burgdorferi *cells in the second exponential phase when cultured in the absence of free GlcNAc [10]. Since we have shown the *rpoS *mutant failed to initiate a second exponential phase in the absence of free GlcNAc by 381 h (Fig. 1), we hypothesized that the *rpoS *mutant may not exhibit a second exponential phase because RpoS is involved, directly or indirectly, in the regulation of *chbC *transcription. To test this hypothesis, RNA was collected from B31-A, A74 and WC12 at various times during growth in media lacking free GlcNAc, and the expression of *chbC *was evaluated by real time quantitative reverse transcription PCR (qRT-PCR) (Fig. 3).

Cells were collected for RNA extraction at 44 h after initiation of the growth experiment and at various time points thereafter. Fold differences in *chbC *expression were calculated by comparing expression at the various time points to the expression at 44 h (Fig. 3). This time point was chosen as the baseline as cells are still in the first exponential phase and in the presence of residual free GlcNAc or chitobiose from yeastolate or rabbit serum (see below). Prior expression studies conducted by Tilly *et al *[10] demonstrated that *chbC *levels remain low in the presence of free GlcNAc. In addition, we evaluated the expression of *chbC *in cells cultured in the absence of GlcNAc and supplemented with high or low concentrations of chitobiose (data not shown). As in complete a medium, *chbC *expression levels remained low until chitobiose was exhausted and cells became starved for GlcNAc (data not shown).

**Figure 4:**
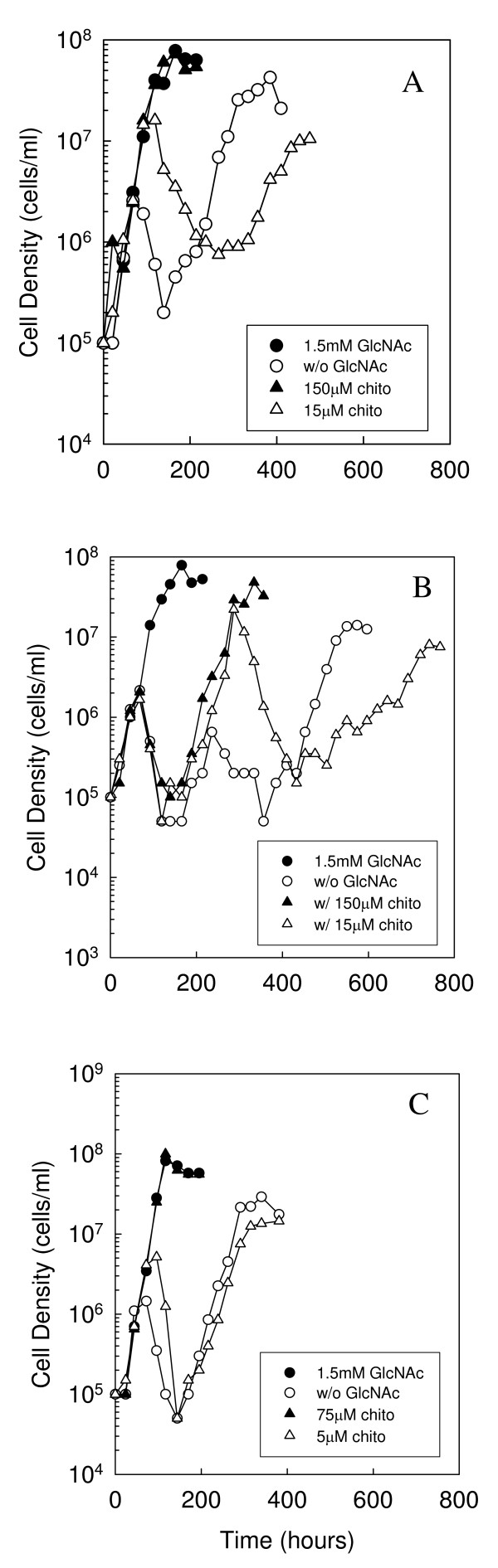
**Mutation of *rpoS *delays chitobiose utilization**. Growth of *B. burgdorferi *strains (A) B31-A (WT), (B) A74 (*rpoS *mutant) and (C) WC12 (*rpoS *complemented mutant) in BSK-II lacking GlcNAc (open circle) and supplemented with 1.5 mM GlcNAc (closed circle), or high (closed triangle, 75 μM or 150 μM) or low (open triangle, 5 μM or 15 μM) concentrations of chitobiose. Late-log phase cells were diluted to 1.0 × 10^5 ^cells ml^-1 ^in the appropriate medium, incubated at 33°C, and enumerated daily as described in the Methods. This is a representative experiment that was repeated three times.

In wild-type cells, *chbC *levels increased by 22-fold at 195 h just as cells entered the second exponential phase. Expression of *chbC *peaked with a 44-fold increase at 239 h, before declining as cells entered stationary phase. In contrast, expression of *chbC *in the *rpoS *mutant did not change from baseline levels for the first 340 h. However, expression did increase by 6-fold at 381 h, which may correspond to this strain beginning to enter a second exponential phase after 400 h (Fig. 4B). When expression of *chbC *was evaluated in the *rpoS *complemented mutant (WC12), levels increased as cells entered the second exponential phase similar to that observed in the wild type. A 27-fold increase was observed at 216 h as cells started to grow in the second exponential phase, and expression peaked with a 40-fold increase at 239 h before declining as cells entered stationary phase. Statistical analysis was performed to determine the significance of *chbC *expression between B31-A and A74 and between WC12 and A74, and fold differences were determined to be statistically significant between 216 and 340 h (p < 0.001).

### Effect of RpoS on chitobiose utilization

In order to evaluate the importance of RpoS in chitobiose utilization, we compared growth of B31-A, A74 and WC12 in BSK-II lacking GlcNAc and supplemented with low (5 μM or 15 μM) or high (75 μM or 150 μM) concentrations of chitobiose (Fig. 4A–C). As expected from the previous study [10], B31-A exhibited a single exponential phase when cultured with a high concentration (150 μM) of chitobiose, reaching a peak cell density of 7.8 × 10^7 ^cells ml^-1 ^by 166 h (Fig. 4A). In contrast, when B31-A was cultured with ten-fold less chitobiose (15 μM) biphasic growth was observed. Biphasic growth in the presence of 15 μM chitobiose differed from that observed in cells cultured without both free GlcNAc and chitobiose, as cells in the first exponential phase grew to a density that was 6.3-fold higher in the presence of low levels of chitobiose (1.6 × 10^7 ^cells ml^-1^) compared to no added chitobiose or GlcNAc (2.5 × 10^6 ^cells ml^-1^).

To determine if RpoS is required for chitobiose utilization, we cultured A74 in BSK-II without GlcNAc and supplemented with low (15 μM) or high (150 μM) concentrations of chitobiose (Fig. 4B). In contrast to the wild type, the *rpoS *mutant was initially unable to utilize chitobiose at either concentration, as cells only grew to 2.0 × 10^6 ^cells ml^-1 ^before blebbing and entering a death phase. At about 200 h cells cultured in both low and high concentrations of chitobiose entered a second exponential phase. In comparison to the wild type, these results suggest that RpoS is important for chitobiose utilization, as the *rpoS *mutant cultured in the absence of free GlcNAc and supplemented with chitobiose could not initially utilize free chitobiose as a source of GlcNAc. A74 cultured in the presence of 150 μM chitobiose reached a peak cell density after the second exponential phase of 4.8 × 10^7 ^cells ml^-1 ^by 334 h before entering stationary phase. In contrast, A74 cultured in the presence of 15 μM chitobiose reached a peak density of 2.2 × 10^7 ^cells ml^-1 ^at 287 hours before blebbing and entering a second death phase in which cell numbers declined to 1.5 × 10^5 ^cells ml^-1^. At 525 h, cells cultured with 15 μM chitobiose entered a third exponential growth phase, and grew to a peak cell density of 1.4 × 10^7 ^cells ml^-1 ^before entering stationary phase. With exception of the first death phase, these results are consistent with those obtained for the wild type cultured in 15 μM chitobiose, and further support our hypothesis that the source of GlcNAc during growth in the second exponential phase in the wild type, and the third exponential phase in the *rpoS *mutant, is not free chitobiose.

Inspection of A74 growth without GlcNAc and without chitobiose revealed triphasic growth similar to, though less pronounced than, that observed in A74 cultured in 15 μM chitobiose (Fig. 4B). As expected, cells reached a density of 2.2 × 10^6 ^cells ml^-1 ^in the first exponential phase before blebbing and entering the first death phase. At 189 h the *rpoS *mutant entered a second exponential phase, and reached a peak density of 6.5 × 10^5 ^cells ml^-1 ^at 236 h. This second exponential phase corresponds to that observed when A74 is grown in the presence of chitobiose, suggesting there is a small amount of free chitobiose present in BSK-II. After a second death phase, cells entered a third exponential phase at 385 h and reached a peak cell density of 1.4 × 10^7 ^cells ml^-1 ^at 574 h before entering stationary phase. This final exponential phase may explain the slight increase in *chbC *expression observed by qRT-PCR at 381 h (Fig. 3).

To confirm the role of RpoS in chitobiose utilization, we evaluated the growth of the *rpoS *complemented mutant, WC12, in BSK-II without GlcNAc and supplemented with low (5 μM) and high (75 μM) concentrations of chitobiose (Fig. 4C). As expected, growth of the complemented mutant was similar to that observed for the wild type. In the presence of high concentrations of chitobiose, the complemented mutant exhibited a single exponential phase and reached a peak density of 8.2 × 10^7 ^cells ml^-1 ^by 117 h before entering stationary phase. In contrast, when the complemented mutant was cultured with the low concentration (5 μM) of chitobiose biphasic growth was observed. In the first exponential phase cells grew to a peak cell density of 5.2 × 10^6 ^cells ml^-1 ^at 96 h before blebbing and entering a death phase. This was a 3.5-fold higher cell density than that observed in the complemented mutant cultured without GlcNAc and without chitobiose. At 170 h the complemented mutant entered a second exponential phase, which peaked at a cell density of 1.5 × 10^7 ^cells ml^-1^. These results lend further support to the hypothesis that RpoS plays a role in the utilization of chitobiose.

### Effect of RpoN on chitobiose utilization

**Figure 5:**
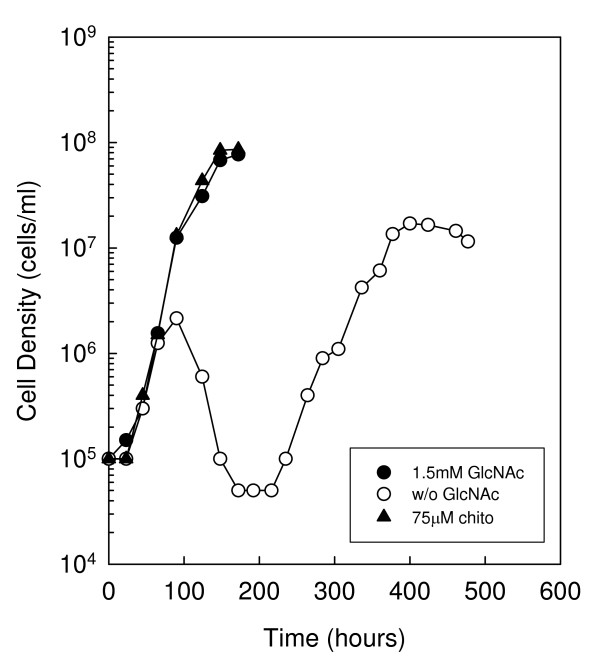
**RpoN is not required for chitobiose utilization**. Growth of *B. burgdorferi *strain RR22 in BSK-II lacking GlcNAc and supplemented with 75 μM chitobiose. Late-log phase cells were diluted to 1.0 × 10^5 ^cells ml^-1 ^in the appropriate medium (closed circle, 1.5 mM GlcNAc; open circle, No addition, i.e. without GlcNAc; closed triangle, 75 μM chitobiose), incubated at 33°C and enumerated daily as described in the Methods. This is a representative experiment that was repeated three times.

Several reports have demonstrated that under certain conditions *rpoS *expression is regulated directly by RpoN [19,20]. To determine if RpoN plays a role in chitobiose utilization, we generated an *rpoN *mutant in the B31-A background (RR22) and evaluated its growth in BSK-II lacking GlcNAc and supplemented with a high concentration of chitobiose (Fig. 5). In the complete medium, RR22 exhibited growth similar to the wild type, reaching a peak cell density of 7.7 × 10^7 ^cells ml^-1 ^by 172 hours. In BSK-II lacking GlcNAc RR22 exhibited biphasic growth similar to the wild type, as initiation of the second exponential phase occurred at 235 hours. When cultured in a medium lacking GlcNAc and supplemented with 75 μM chitobiose RR22 exhibited only one exponential phase, and reached a peak cell density of 8.6 × 10^7 ^cells ml^-1 ^by 172 h. These results suggest RpoN is not necessary for chitobiose utilization. It is important to note that growth curves of the *rpoN *mutant were conducted in parallel with the wild type, *rpoS *mutant and *rpoS *complemented mutant growth experiments (Fig. 4).

### Identification of the *chbC *transcriptional start site and promoter analysis

**Figure 6:**
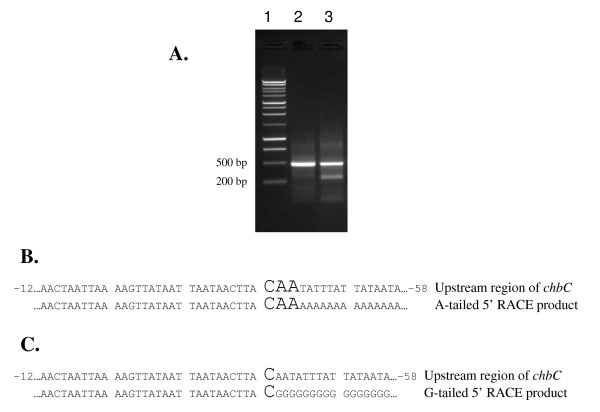
**Determination of the *chbC *transcriptional start site**. The *chbC *transcriptional start site was determined by 5' RACE analysis. (A) One percent TAE agarose gel of the 5' RACE products. A 1 kb ladder was used as a size standard (lane 1) for comparison of 5' RACE products (lane 2, dA-tailed product; lane 3, dG-tailed product). (B) DNA sequence of the dA-tailed 5' RACE product showing the ambiguous *chbC *transcriptional start site (enlarged font). (C) DNA sequence of the dG-tailed 5' RACE product showing the *chbC *transcriptional start site (enlarged font). Sequences were determined using the anti-sense primer BBB04 5' RACE R2.

The results above demonstrate that RpoS regulates the expression of *chbC*, at least partially, and is important in chitobiose utilization *in vitro*. To determine if the *chbC *gene has a promoter similar to other RpoS-dependent genes, we performed 5' RACE to identify the transcriptional start site of *chbC *and compared the promoter region with previously described RpoD, RpoS and RpoN-dependent promoter sequences in *B. burgdorferi*. Total RNA was extracted from B31-A and used to generate *chbC*-specific cDNA in a reverse transcription reaction. The cDNA was purified and a homopolymeric dA-tail was added. Subsequent PCR with the oligo dT-anchor primer and a nested *chbC*-specific primer (BBB04 5' RACE R2) resulted in an approximate 410 bp product (Fig. 6A; lane 2). The PCR product was sequenced, and the transcriptional start site was determined to be between 42 and 44 base pairs upstream of the translational start site (Fig. 6B). To resolve this ambiguity *chbC*-specific cDNA was tailed with dGTP, and PCR was carried out using the oligo dC-anchor primer and the nested *chbC*-specific primer. Separation of this PCR by gel electrophoresis revealed two products that were approximately 250 and 410 base pairs (Fig. 6A; lane 3). The bands were gel extracted and sequenced. Sequence analysis of the lower band showed this product was from mispriming of the oligo dC-anchor primer to three guanosines located 160 to 162 base pairs downstream of the *chbC *translational start site (data not shown). Comparison of the sequences from the upper dG-tailed product (Fig. 6C) and the dA-tailed product (Fig. 6B) revealed the *chbC *transcriptional start site 42 base pairs upstream of the translational start site.

**Figure 7:**
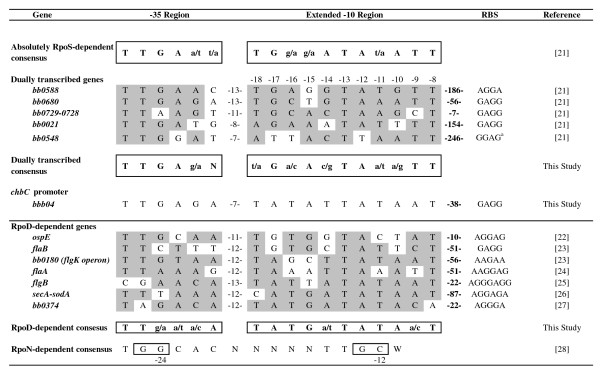
**Identification of the *chbC *promoter**. Comparison of the *chbC *promoter region with consensus promoter sequences for RpoS, dually transcribed, and RpoD-dependent genes. Nucleotide positions of promoters from dually transcribed and RpoD-dependent genes that match their respective consensus sequence are highlighted, and consensus sequences are boxed. The RpoN consensus sequence is also included for comparison, and the highly conserved GG and GC doublets at the -24 and -12 regions are boxed.

Identification of the *chbC *transcriptional start site allowed us to identify the -10 and -35 promoter regions by visual inspection of the upstream sequence (Fig. 7). Further analysis of the promoter region was conducted by comparing the putative *chbC *promoter to previously described *B. burgdorferi *promoters controlled by RpoD, RpoS and RpoN (Fig. 7). Recently, Caimano *et al *[21] evaluated the RpoS regulon in *B. burgdorferi *by microarray and qRT-PCR expression analysis and identified genes that were absolutely RpoS-dependent as well as genes that were dually transcribed by RpoS and at least one of the other sigma factors in *B. burgdorferi*. Analysis of the promoter region from ten absolutely RpoS-dependent genes allowed them to identify a putative RpoS consensus -10 and -35 sequence (Fig. 7). In addition, they attempted to identify the promoter regions for 10 dually transcribed genes, but were only able to find putative promoter elements for five of the genes which were highly similar to the consensus sequence generated from the absolutely RpoS-dependent genes. We used these five putative promoters to generate a dually transcribed -10 and -35-consensus sequence for comparison to our newly identified *chbC *promoter region (Fig. 7), as results presented above strongly suggest that this gene is dually regulated by RpoS and RpoD. Additionally, we generated a consensus RpoD-dependent promoter sequence for comparison (Fig. 7) based on seven genes identified in the literature [22-27].

Comparison of the *chbC *promoter region to the absolutely RpoS-dependent consensus or the dually transcribed consensus sequences revealed the same differences in four of the eleven extended -10 positions. In contrast, there was only a one base difference between the *chbC *extended -10 and that of the RpoD consensus promoter (Fig. 7). As expected, the -35 consensus for the RpoS and RpoD-dependent promoters were very similar, and the sequence for the *chbC *-35 region only differed by one base when compared to both consensus sequences. Of note, the spacing between the end of the extended -10 and the beginning of the -35 sequences for the dually transcribed genes ranged from 7 to 13 bases, whereas for RpoD-dependent genes the spacing ranged from 11 to 13 bases. The predicted spacing between the extended -10 and -35 of the *chbC *promoter was 7 bases, which is similar to at least 2 dually transcribed genes. Finally, the consensus RpoN-dependent sequence is shown for comparison, and there is no evidence of the GG and GC doublets in the highly conserved -24/-12 regions of the *chbC *promoter that are typically observed in genes directly controlled by RpoN (Fig. 7) [28].

### Growth of *B. burgdorferi *without yeastolate

**Figure 8:**
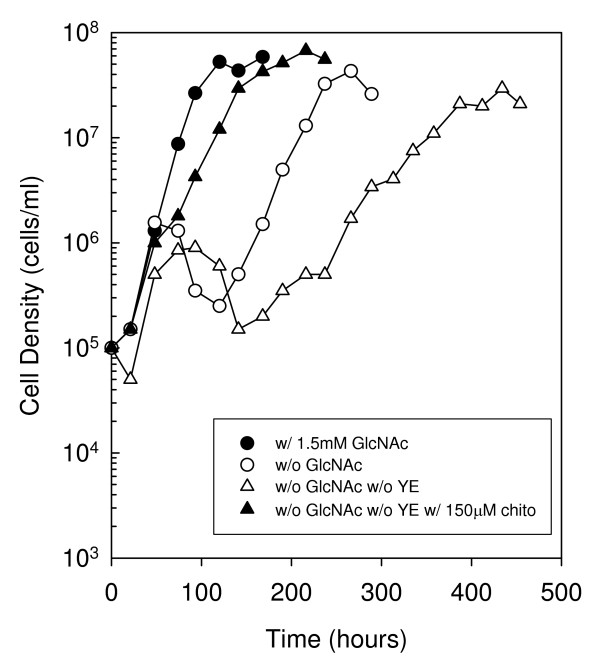
**Growth of *B. burgdorferi *strain B31-A in BSK-II without GlcNAc and yeastolate, and supplemented with 150 μM chitobiose**. Late-log phase cells were diluted to 1.0 × 10^5 ^cells ml^-1 ^in the appropriate medium (closed circle, 1.5 mM GlcNAc, with Yeastolate; open circle, without GlcNAc, with Yeastolate; open triangle, without GlcNAc, without Yeastolate; closed triangle, without GlcNAc, without Yeastolate, with 150 μM chitobiose), incubated at 33°C, and enumerated daily as described in the Methods. This is a representative experiment that was repeated three times.

Yeastolate, a component of BSK-II, is the water-soluble portion of autolyzed *Sacchromyces cerevisiae*, and contains a mixture of peptides, amino acids, vitamins and simple and complex carbohydrates. As the preparation is derived from yeast it likely contains chitobiose and/or longer GlcNAc oligomers available to *B. burgdorferi *as a source of GlcNAc. Previously, Tilly *et al *[10] suggested that yeastolate was the source of GlcNAc for growth of the wild type in the second exponential phase, as cells failed to exhibit a second exponential phase by 250 hours when cultured without GlcNAc and without yeastolate. However, we hypothesized that yeastolate may not be the source of GlcNAc during the second exponential phase, since *B. burgdorferi *can utilize chitobiose in the absence of free GlcNAc to maintain normal growth and reach optimal cell densities in a single exponential phase. To test this hypothesis we followed growth of wild-type cells in BSK-II without free GlcNAc and yeastolate for an extended period of time (Fig. 8). In contrast to the previous report, biphasic growth was observed in cells cultured without GlcNAc and yeastolate, suggesting that the source of GlcNAc for growth in the second exponential phase was not chitobiose or GlcNAc oligomers present in yeastolate. Additionally, cells cultured without GlcNAc and yeastolate reached a peak cell density of 9.0 × 10^5 ^cells ml^-1 ^in the first exponential phase prior to entering a death phase at 93 hours. This was approximately 2-fold lower than that reached in cells cultured without free GlcNAc only. This suggests that cells cultured in the absence of free GlcNAc with yeastolate exhausted the residual free GlcNAc and/or GlcNAc oligomers present in yeastolate before declining in density. A second exponential phase was observed in the culture without GlcNAc and yeastolate beginning at 266 hours, reaching a peak cell density of 3.0 × 10^7 ^cells ml^-1 ^at 434 hours before entering stationary phase. Furthermore, when chitobiose was added to cells cultured without GlcNAc and yeastolate a single exponential phase was observed, though the growth rate was slightly reduced. Taken together, these data suggest that the source of GlcNAc in the second exponential phase is due to components in BSK-II other than yeastolate.

## Discussion

In the present study we evaluated the role of RpoS and RpoN on biphasic growth and chitobiose utilization in *B. burgdorferi *cells cultured in the absence of free GlcNAc. RpoS and RpoN are the only two alternative sigma factors encoded by *B. burgdorferi*, and have been shown to play key roles in the regulation of genes necessary for colonization of both the tick vector and mammalian host [17-19,29]. A previous report demonstrated that biphasic growth in a medium lacking free GlcNAc is dependent on *chbC *expression, as *chbC *transcript levels in wild-type cells were increased during the second exponential phase [10]. We added to those results here by demonstrating that RpoS in the B31-A background regulates biphasic growth, as initiation of the second exponential phase was delayed by more than 200 h in the *rpoS *mutant when compared to the wild type and *rpoS *complemented mutant (Figs. 1 and 4A–C). Our results also suggest the delay in the *rpoS *mutant is due, at least in part, to its inability to up regulate *chbC *before 340 h during GlcNAc starvation (Fig. 3). In contrast, *chbC *transcript levels increased in the wild type and *rpoS *complemented mutant, corresponding to the initiation of a second exponential phase in these strains (Fig. 3). Taken together, these results confirm the requirement for *chbC *expression during growth in the second exponential phase [10], and suggest that RpoS regulates biphasic growth in media lacking free GlcNAc through regulation of *chbC *transcription.

Tilly *et al *[10] previously demonstrated that wild-type *B. burgdorferi *can efficiently transport and utilize chitobiose in the absence of free GlcNAc to grow to optimal cell densities in one exponential phase, with optimal growth occurring at chitobiose concentrations ≥ 18 μM. We confirmed those observations and also demonstrated that *B. burgdorferi *exhibits biphasic growth when cultured with low concentrations (≤ 15 μM) of chitobiose (Fig. 4A). This observation suggests that free chitobiose, and potentially longer free GlcNAc oligomers, are not the source of GlcNAc for growth in the second exponential phase, as was previously suggested [10]. In fact, growth of the wild type without GlcNAc but supplemented with longer GlcNAc oligomers, chitotriose and chitohexose, results in optimal cell densities and only one exponential phase (Rhodes and Nelson, manuscript in preparation). This observation suggests that *B. burgdorferi *employs one or more enzymes for the breakdown of longer GlcNAc oligomers, and that this mechanism of obtaining sequestered (or bound) GlcNAc in the form of chitin is turned on during the first exponential phase.

Chitin and chitobiose may serve as an important nutrient source during the tick molt, as the peritrophic membrane encasing the blood meal is turned over and GlcNAc oligomers are released [8]. Previous laboratory studies by Tilly *et al *[11] demonstrated that *chbC *is not necessary for *B. burgdorferi *to complete an infectious cycle, leading them to suggest that the genome is still evolving and retains non-essential functional genes. However, we argue that selective pressure must be involved in the retention of this three component PTS, as it is also found in other *Borrelia *species (*garinii *and *afzelli*) that cause Lyme borreliosis and to our knowledge there has not been a strain isolated in which this transport system is not present. This may be an instance in which mixed infection studies would be appropriate to determine the competitive index (i.e. degree of virulence attenuation) for the *chbC *mutant as compared to the wild type.

To further demonstrate that free chitobiose or longer GlcNAc oligomers are not the source of GlcNAc in the second exponential phase, we followed the growth of cells in a medium lacking free GlcNAc and yeastolate (Fig. 8). Yeastolate is the only component of BSK-II that may contain GlcNAc oligomers, as it is derived from an organism with a chitinous cell wall. Tilly *et al *[10] previously reported that there was no second exponential phase by 250 hours when cells were cultured without free GlcNAc and yeastolate, and therefore, suggested that chitobiose and possibly other GlcNAc oligomers present in yeastolate may be the source of GlcNAc for growth in the second exponential phase. However, our results demonstrate that wild-type cells do exhibit a second exponential phase in the absence of free GlcNAc and yeastolate, and reach a peak cell density in the second exponential phase by 434 hours. This result suggests yeastolate alone is not the source of GlcNAc for growth in the second exponential phase.

One possible explanation for the biphasic growth observed in the absence of free GlcNAc or limiting amounts of chitobiose is that a mutation has occurred allowing for the outgrowth of a mutant population. A previous report from Tilly *et al *[10] suggested this was not the case as cells back-diluted from the second exponential phase into a medium without GlcNAc still exhibited biphasic growth. However, in that experiment cells that were back-diluted grew almost 10-fold higher in the first exponential phase compared to cells in the first exponential phase from the original culture. This suggests the back-diluted cells were now able to utilize a GlcNAc-containing medium component that they were not previously able to use. In fact, unpublished data from our laboratory supports the hypothesis (Rhodes and Nelson, manuscript in preparation) that neopeptone (an enzymatic digest of protein) and rabbit serum supply GlcNAc sequestered in the form of glycoproteins or proteoglycans that *B. burgdorferi *can acquire and utilize for growth in the second exponential phase. Numerous reports have demonstrated adhesion of *B. burgdorferi *to mammalian cells through the binding of glycoproteins such as fibronectin [30], glycosaminoglycans such as heparin sulfate [31], and proteoglycans such as decorin [32]. The ability to bind these substrates brings the spirochetes into close proximity with bound GlcNAc, and may represent a valuable source of this sugar when free GlcNAc or GlcNAc oligomers are not available. A deglycosylation mechanism has recently been described in *Streptococcus pneumoniae*, in which exoglycosidases sequentially remove sugar residues from host glycoproteins [33]. We suggest that *B. burgdorferi *may employ similar mechanisms by which they can release and utilize bound GlcNAc from host-derived glycoproteins, glycosaminoglycans and/or proteoglycans. Results described above suggest that some, if not all, of the GlcNAc imported into the cell in the second exponential phase comes in the form of chitobiose. The proposed mechanism for obtaining GlcNAc from glycoproteins would be consistent with this as the oligosaccharide portion of N-linked glycoproteins is attached to the amino acid asparagine through chitobiose [34]. This core chitobiose residue as well as others present throughout the oligosaccharide moiety may be sources of GlcNAc for *B. burgdorferi *during growth in the second exponential phase.

A second possible explanation for biphasic growth is that it is the result of scavenging of GlcNAc released from dead *B. burgdorferi *cells. While it cannot be ruled out that some growth in the second exponential phase may be due to scavenging of GlcNAc from dead cells, it is unlikely that all of the growth is due to scavenging as the peak cell density in the second exponential phase is > 5-fold higher than the cell density reached in the first exponential phase. Therefore, there would not be a sufficient amount of GlcNAc present in dead cells to account for the cell density reached in the second exponential phase. This point was made previously by Tilly *et al *[10].

Since our experiments with the A74 *rpoS *mutant strongly suggest that RpoS plays an important role in biphasic growth and *chbC *expression in the B31-A background in the absence of free GlcNAc, we also evaluated the ability of the *rpoS *mutant to utilize free chitobiose. Unlike the wild type (Fig. 4A) and *rpoS *complemented mutant (Fig. 4C), the *rpoS *mutant could not utilize chitobiose initially and did not show chitobiose-stimulated growth until 200 h (Fig. 4B). The *rpoS *mutant began a second exponential phase at 200 h with or without the addition of free chitobiose (Fig. 4B), and triphasic growth was observed in the absence of free GlcNAc and chitobiose. These results indicate there is a small amount of free chitobiose present in BSK-II, most likely as a component of the yeastolate or rabbit serum. The addition of a low (15 μM) concentration of free chitobiose also resulted in triphasic growth (Fig. 4B), but in this case growth in the second exponential phase was more than 30-fold higher when compared to culturing the *rpoS *mutant in the absence of free GlcNAc and chitobiose. Together, these results strongly suggest that RpoS, at least partially, regulates chitobiose utilization, and further demonstrate that free chitobiose is not the source of GlcNAc in the second exponential phase of the wild type or the third exponential phase of the *rpoS *mutant.

Previous reports have demonstrated that a RpoN-RpoS cascade regulates the expression of outer membrane lipoproteins, such as OspC and Mlps (multicopy lipoproteins), in *B. burgdorferi *[19,20,35]. Therefore, we generated a high-passage B31-A *rpoN *mutant to determine if RpoN is involved in the regulation of chitobiose utilization. We were surprised to discover that our *rpoN *mutant behaved similarly to the wild type, exhibiting only one exponential phase when cultured without GlcNAc and supplemented with 75 μM chitobiose (Fig. 5). This result suggests that RpoN is not involved in the utilization of free chitobiose, and therefore this pathway appears to be regulated by only RpoS and RpoD. While our results do seem to challenge the well established RpoN-RpoS paradigm in *B. burgdorferi*, our experiments were performed under different conditions. Typically, RpoS-dependent genes are evaluated *in vitro *in a temperature-dependent manner where cultures are shifted from 23°C to 35°C [17,21]. However, our experiments were conducted exclusively at 33°C as we observed a change in the phenotype of the *rpoS *mutant at this temperature (biphasic growth and decreased *chbC *expression) that could be restored when the wild-type gene was re-introduced on a plasmid. In addition, we are not the first group to demonstrate RpoS regulation in the absence of RpoN. Despite evidence of direct regulation of *rpoS *by RpoN [20], Fisher *et al *[18] also showed that RpoS can regulate genes in the absence of RpoN. It is interesting to note that in this microarray study BBB05 and BBB06 (*chbA *and *chbB*, respectively) declined by 40–50% in a *rpoN *mutant. No changes in BBB04, BBB05, or BBB06 transcription were reported for their *rpoS *mutant. However, in that study, Fisher *et al *[18] did not starve cells for GlcNAc, a technique that in our hands results in a modest 2-fold increase in *rpoS *transcript levels (data not shown), and a corresponding increase in *chbC *expression (Fig. 3). Additionally, Lybecker and Samuels [36] recently demonstrated that two *rpoS *transcripts exist, a shorter RpoN-regulated transcript previously identified by Smith *et al*. [20] that predominates at high cell density, and a longer transcript that does not possess the canonical RpoN-dependent promoter whose translation is regulated by the small RNA (sRNA) DsrA_Bb _at low cell density.

Our physiological and molecular data evaluating chitobiose utilization (Fig. 4) and *chbC *expression (Fig. 3) in the wild type versus the *rpoS *mutant strongly suggests that RpoD and RpoS both regulate chitobiose transport. To determine if the *chbC *gene has a promoter similar to other RpoS-dependent genes we identified the transcriptional start site (Fig. 6) and the putative *chbC *promoter (Fig. 7). While not conclusive, it is possible that regulation of *chbC *by RpoS is through direct binding to the promoter region as the spacing between the -10 and -35 consensus sequences is similar to that of two of the dually transcribed promoters (Fig. 7). On the other hand, the sequence of the extended -10 *chbC *promoter element is more like that of the predicted RpoD consensus, and it has been shown that the extended -10 element plays a significant role in sigma factor selectivity in *B. burgdorferi *[37]. Therefore, it cannot be ruled out that RpoS regulates *chbC *expression indirectly through an unknown regulator, rather than through direct binding and transcription from the *chbC *promoter.

## Conclusion

In this study we used a physiologic and molecular approach to demonstrate that chitobiose utilization and *chbC *expression are dually regulated by RpoD and RpoS. We determined the *chbC *transcriptional start site, and identified the putative promoter region. Finally, we provided evidence that the second exponential phase observed in cells cultured in the absence of free GlcNAc is not due to components found in yeastolate, and suggest that the source of GlcNAc in the second exponential phase is sequestered in components of serum and/or neopeptone.

## Methods

### Bacterial strains and culture conditions

Wild-type *B. burgdorferi *strain B31-A and *rpoS *mutant strain A74 were generously provided by Patricia Rosa [38]. All strains were routinely cultured in modified BSK-II medium supplemented with 7% rabbit serum (Invitrogen Corp., Carlsbad, CA) [6]. BSK-II was modified by the replacement of 10× CMRL-1066 with 10× Media 199 (Invitrogen Corp.). The *rpoS *mutant (A74) and complemented *rpoS *mutant (WC12) were maintained under selection in BSK-II with either coumermycin A1 (0.5 μg ml^-1^; A74) or both coumermycin A1 (0.5 μg ml^-1^) and kanamycin (340 μg ml^-1^; WC12). The *rpoN *mutant (RR22) was maintained under selection in BSK-II with erythromycin (0.6 μg ml^-1^). See Table 1 for a summary of strains and plasmids used in this study.

**Table 1:** Strains and Plasmids

Strain or Plasmid	Genotype and Description	Reference
Strains		
*B. burgdorferi*		
B31-A	High passage non-infectious wild-type	[38]
A74	Coum^R^; B31-A *rpoS *mutant	[38]
WC12	Coum^R ^Kan^R^; A74 complemented with *rpoS*_*Bb*_/pCE320	This study
297 *rpoN*	Ery^R^; 297 *rpoN *mutant	[19]
RR22	Ery^R^; B31-A *rpoN *mutant	This study
		
*E. coli*		
DH5α	*supE*44 F^- ^Δ*lac*U169 (ϕ80*lac*Z ΔM15) *hsdR*17 *relA*1 *endA*1 *gyrA*96 *thi*-1* relA*1	[40]
		
Plasmids		
*rpoS*_*Bb*_/pCE320	Kan^R ^Zeo^R^; P_nat_-*rpoS*	[17]
pBB0450.1	Amp^R ^Ery^R^; *ermC*::*rpoN*	This study

### Growth Curves

For growth experiments, late-log phase cells (~5.0 × 10^7 ^cells ml^-1^) were back-diluted to 1.0 × 10^5 ^cells ml^-1 ^in 12 ml of BSK-II lacking GlcNAc or yeastolate, or lacking both GlcNAc and yeastolate. Typically, 12–24 μl of culture was inoculated into 12 ml of fresh medium; therefore, minimal amounts of nutrients were transferred with the inoculum. Cultures were supplemented with 1.5 mM GlcNAc (US Biochemical, Corp., Cleveland, OH), a low concentration of chitobiose (5 or 15 μM; V-Labs, Inc., Covington, LA) or a high concentration of chitobiose (75 or 150 μM). All growth experiments were conducted at 33°C in the presence of 3% CO_2_. Cells were enumerated daily by darkfield microscopy using a Petroff-Hausser counting chamber (Hausser Scientific, Horsham, PA). Specifically, 2.5 μl of undiluted culture was transferred to the counting chamber and cells were counted in all 25 squares. Once cells reached a density >1.0 × 10^7 ^cells ml^-1 ^the culture was diluted 1:10 in BSK-II prior to enumeration. Each growth curve is representative of at least three independent trials. Growth data from independent experiments could not be pooled due to the length of the experiments and the different times at which bacteria were enumerated.

### Complementation of the *B. burgdorferi rpoS *mutation

A complemented *rpoS *mutant of A74 was generated using *rpoS*_Bb_/pCE320 (donated by Justin Radolf) [17], which consists of the wild-type *rpoS *gene under the control of its natural promoter. The plasmid contains a kanamycin resistance gene under the control of the constitutive *flgB *promoter, and was maintained in *E. coli *DH5α grown in lysogeny broth (LB; 1% tryptone, 0.5% yeast extract, 1% NaCl) supplemented with kanamycin (50 μg μl^-1^). The QIAprep Spin Mini Kit (Qiagen, Inc., Valencia, CA) was used to extract plasmid according to the manufacturer's instructions. Plasmid *rpoS*_Bb_/pCE320 was concentrated to greater than 1 μg μl^-1^, and 10 μg of plasmid was transformed into competent A74. Cells from the transformation reaction were resuspended in 10 ml of BSK-II containing 20 μg ml^-1 ^phosphomycin, 50 μg ml^-1 ^rifampicin and 2.5 μg ml^-1 ^amphotericin B (Antibiotic Mixture for *Borrelia *100x; Sigma-Aldrich, St. Louis, MO), and allowed to recover for 18–24 h before plating in BSK-II containing kanamycin (340 μg ml^-1^) according to the protocol of Samuels *et al *[39]. Kanamycin resistant colonies, appearing approximately 10–14 days after plating, were screened for the presence of the complementation plasmid by PCR using primers BB0771 F1 and BB0771 R1 2. A positive clone was chosen for further experiments and designated WC12.

### Construction of the *rpoN *mutant in B31-A

A *B. burgdorferi *297 *rpoN *mutant strain (donated by Michael Norgard) [19], in which *rpoN *was interrupted by the insertion of an erythromycin resistance gene, was maintained in BSK-II containing erythromycin (0.6 μg ml^-1^). Genomic DNA was extracted from the 297 *rpoN *mutant using the DNeasy Tissue Kit (Qiagen, Inc.) following the manufacturer's instructions. Primers BB0450 mutF1 and BB0450 mutR1 (Table 2) were used to PCR amplify *rpoN*::*ermC *and flanking DNA from 297 *rpoN *mutant genomic DNA. The PCR product (~4.4 kb) was TA cloned into the pGEM T-Easy vector (Promega, Corp., Madison, WI) according to the manufacturer's instructions, and the ligation reaction was transformed into competent *E. coli *DH5α. A transformant containing the plasmid of interest was selected by blue-white screening on LB containing ampicillin (200 μg ml^-1^) and X-gal (40 μg ml^-1^), confirmed by PCR using the BB0450 mutF1 and BB0450 mutR1 primers, and designated pBB0450.1.  See Table 2. The plasmid was extracted and concentrated to greater than 1 μg μl^-1^, and 10 μg were transformed into competent B31-A as described above. Transformants were selected by plating on BSK-II containing erythromycin (0.6 μg/ml) according to the protocol of Samuels *et al *[39]. The mutation in the *rpoN *gene of B31-A was confirmed by PCR using primers flanking the *ermC *insertion site (BB0450 mut confirm F1 and BB0450 mut confirm R1.  See Table 2), and the mutant was designated RR22. In addition, DNA sequence analysis (ABI Prism^® ^3130XL Genetic Analyzer, Applied Biosystems, Forest City, CA) was performed to verify the *rpoN*::*ermC *junctions using primers 5' ermC seq out and 3' ermC seq out.  See Table 2. The University of Rhode Island Genomics and Sequencing Center performed DNA sequencing.

**Table 2:** Oligonucleotide primers

Primer Name	Sequence (5'→3')
BB0771 F1	CTTGCAGGACAAATACAAAGAGGC
BB0771 R1	GCAGCTCTTATTAATCCCAAGTTGCC
BB0450 mutF1	TTCTCCTCTTGGAACCATTCCGGT
BB0450 mutR1	ACCATAACCTACCACGCCCTCAAT
BB0450 mut confirm F1	GGTTCCATAATATGTTCTCCCTTTCTCAG
BB0450 mut confirm R1	CCCAACGCTCGAATTTAAAGACCC
5' ErmC seq out	GGCCTTTTCCTGAGCCGATTTCAAAG
3' ErmC seq out	TTCCTTAAAACATGCAGGAATTGACG
*chbC *F	GGGAATTCAGCCCAATTCATGGTTTCC
*chbC *R	GGCGGAACAGACTCTGGAAGCTTAAT
BBB04 5'RACE R1	GCTACAATTGAAAGCGCAACAACAGG
Oligo(dT) AP	GACCACGCGTATCGATGTCGACTTTTTTTTTTTTTTTTV
Oligo(dC) AP	GACCACGCGTATCGATGTCGACCCCCCCCCCCCCCCCC
BBB04 5'RACE R2	AGCAGCATCTCCACCGTAAGGTAT

### RNA extraction

Cells were harvested at various times during growth by centrifugation (10,000 × *g*, 12 min, 4°C). Pellets were resuspended in 500 μl of BSK-II lacking GlcNAc and transferred to 2 ml microcentrifuge tubes. One ml of Bacteria RNAProtect (Qiagen, Inc.) was added and mixed by vortexing. Cells were incubated for 5 min at room temperature, and then centrifuged for 10 min at 5,000 × *g*. Pellets were stored at -80°C for up to 4 weeks prior to RNA extraction. RNA was extracted using the RNeasy Mini kit (Qiagen, Inc.) according to the manufacturer's instructions. RNA was DNase-treated with RQ1 RNase-free DNase (Promega Corp.), and RNasin (Promega Corp.) was added according to the manufacturer's instructions. Protein from the DNase reaction was removed using the RNeasy Mini kit according to the RNA Cleanup protocol supplied by the manufacturer. RNA concentration (OD_260_) and purity (OD_260_/OD_280_) were determined by UV spectrophotometry. RNA integrity was evaluated by gel electrophoresis. Specifically, 2 μg of each sample was separated on a 1% agarose gel and the intensity of the 16S and 23S ribosomal RNA bands was determined. RNA was stored at -80°C for subsequent gene expression analysis.

### Real-time quantitative reverse transcription-PCR (qRT-PCR)

qRT-PCR was performed using the Mx4000 or Mx3005P Multiplex Quantitative PCR System and the Brilliant SYBR Green Single-Step qRT-PCR Master Mix Kit (Stratagene, La Jolla, CA) according to the manufacturer's instructions. A standard curve (10^1 ^to 10^7 ^copies per reaction) was generated using a purified *chbC *PCR product as the template. The following primers were used for all reactions: forward primer *chbC *F and reverse primer *chbC *R. Reactions (25 μl) containing 10 ng of total RNA were run under the following conditions: 1 cycle of 50°C for 30 min and 95°C for 15 min, followed by 40 cycles of 95°C for 30 s and 58°C for 30 s 2. Fluorescence was measured at the end of the 58°C step every cycle. Samples were run in duplicate, and all qRT-PCR experiments included both no-reverse transcriptase (RT) and no-template controls. The copy number of *chbC *mRNA in each sample was determined using the MxPro (Stratagene) data analysis software based on the *chbC *standard curve described above. The *chbC *copy number for each sample was normalized based on the total RNA input (10 ng per reaction), and fold differences in *chbC *expression from the initial time point (44 h) were calculated based on the normalized copy numbers.

### Identification of the *chbC *transcriptional start site and promoter analysis

Total RNA was isolated from wild-type *B. burgdorferi *strain B31-A cultured in complete BSK-II as described above. The transcriptional start site was determined using the 2^nd ^Generation 5'/3' RACE Kit (Roche Applied Science; Mannheim, Germany) according to the manufacturer's instructions. Briefly, first-strand cDNA synthesis was carried out in a reverse transcription reaction for 60 min at 55°C using primer BBB04 5' RACE R1 2 and 1 μg of total RNA. The *chbC-*specific cDNA was purified using the High Pure PCR Product Purification Kit (Roche Applied Science). The resulting purified cDNA was split to two reaction tubes and a homopolymeric A or G-tail was added to the 3' end using recombinant terminal deoxynucleotidyl transferase and dATP or dGTP. A PCR product was amplified from each tailed cDNA using the appropriate anchor primer (oligo dT-AP or oligo dC-AP) and the nested gene specific primer BBB04 5' RACE R2 2. The PCR products were subjected to electrophoresis and the bands were gel extracted using the QIAquick PCR Purification Kit (Qiagen). The sequence of the purified PCR products was determined using the BBB04 5' RACE R2 primer and aligned to the upstream sequence of *chbC *to determine the transcriptional start site. Promoter analysis was carried out by visual inspection and comparison of the region upstream of the *chbC *transcriptional start site with previously described RpoD, RpoS and RpoN-dependent promoter sequences in *B. burgdorferi*.

## List of Abbreviations

GlcNAc: N-acetylglucosamine; PTS: phosphotransferase system; RACE: rapid amplification of cDNA ends; BSK-II: Barbour-Stoenner-Kelly medium; Coum^R^: coumermycin A_1 _resistant; Kan^R^: kanamycin resistant; Ery^R^: erythromycin resistant, Zeo^R^: zeocin resistant; YE: yeastolate; chito: chitobiose.

## Authors' contributions

RGR and DRN conceived of the study. RGR performed the growth curve analyses and qRT-PCR, constructed the *rpoN *mutant (RR22) in the B31-A background, determined the transcriptional start site of *chbC*, and drafted the manuscript. WC constructed and confirmed the *rpoS *complemented mutant (WC12). DRN supervised the work and edited the manuscript. All authors read and approved the final manuscript.
